# Activation of p53 and destabilization of androgen receptor by combinatorial inhibition of MDM2 and MDMX in prostate cancer cells

**DOI:** 10.18632/oncotarget.23569

**Published:** 2017-12-15

**Authors:** Harman Chopra, Zara Khan, Jamie Contreras, Herui Wang, Abanob Sedrak, Yan Zhu

**Affiliations:** ^1^ Department of Biological Sciences, St. John's University, Queens, NY 11439, USA

**Keywords:** androgen receptor, p53, MDM2, MDMX, prostate cancer

## Abstract

Castration-resistant prostate cancer (CRPC) frequently develops after initial standard radiation and androgen deprivation therapy, leaving patients with limited further treatment options. Androgen receptor (AR) is a transcription factor that plays a key role in the initiation and progression of prostate cancer. p53, a major tumor suppressor that is rarely mutated in early-stages of prostate cancer, is often deregulated during prostate cancer progression. Here, we report an unusual co-amplification of MDM2 and MDMX, two crucial negative regulators of p53, in CRPC datasets. We demonstrate that combinatorial inhibition of MDM2 and MDMX, with nutlin-3 and NSC207895 respectively, has a profound inhibitory effect on cell proliferation of androgen-responsive, wild-type TP53 gene carrying prostate cancer cells LNCaP and 22Rv1. We further show that the combinatorial inhibition of MDM2 and MDMX not only activates p53, but also decreases cellular levels of AR and represses its function. Additionally, co-expression of MDM2 and MDMX stabilizes AR. Together, our results indicate that combinatorial inhibition of MDM2 and MDMX may offer a novel compelling strategy for prostate cancer therapy.

## INTRODUCTION

Prostate cancer is the most common cancer in men [1]. Since majority of patients progress to castration-resistant prostate cancer (CRPC) with limited treatment options [2–4], new therapies are urgently needed. Prostate cancer initiation and progression are uniquely dependent on the androgen receptor (AR), a hormone-inducible DNA-binding transcription factor that plays a critical role in the development and function of the prostate [5, 6]. Although the requirement for androgen is no longer necessary in CRPR, AR can still potentiate tumor growth and survival, and appears to be the principal accomplice in progression towards complete androgen-independence [4, 7–9]. Thus, activity of the AR remains an important therapeutic target even in advanced stages of the androgen independent CRPC [10–12].

The tumor suppressor p53, the guardian of the genome, serves as one of the major cellular barriers against cancer development [13, 14]. The p53 protein functions as a transcription factor that is activated in response to virtually all cancer-associated stress signals, and regulates genes involved in cell cycle, DNA damage response, apoptosis, and metabolism [14, 15]. MDM2, a major p53 negative regulator, potently inhibits p53 by MDM2-mediated p53 degradation [16, 17], inhibition of p53 transactivation [18], and cytoplasmic translocation of p53 [19]. Amplification of *mdm2* has been observed in more than 10% of human cancers and has been found sufficient to induce tumorigenesis [20–22]. MDMX (also referred to as MDM4), the MDM2 homologue and another crucial negative regulator of p53, inhibits the p53 function mainly by repressing its transcriptional activity [13]. Although MDMX lacks the E3 ubiquitin ligase activity [23], emerging evidence suggests that MDMX can also regulate the stability of p53 through promoting MDM2-mediated degradation through MDM2/MDMX heterodimer formation [24–27]. Overexpression of MDMX has been documented in different types of human cancers [28]. Interestingly, overexpression of MDM2 and MDMX is often mutually exclusive in cancer cells [29], suggesting that dysregulation of either one of the inhibitors is sufficient for p53 inactivation, leading to tumor development. Because the *TP53* gene often remains wild-type in MDM2- or MDMX-overexpressing cancers, it has long been thought that targeting MDM2 or MDMX could restore p53 activity for cancer therapy [28, 30, 31]. Chemotherapeutic drugs that induce p53 as well as small molecules that disrupt the interaction between p53 and MDM2 or MDMX have been shown to induce cell death in prostate cancer cells [32–34]. Additionally, p53 activation has been found to augment the antitumor outcome of androgen ablation in prostate cancer [32].

Here, we report an unusual co-amplification of MDM2 and MDMX in CRPC datasets. We show that nutlin-3 (an MDM2 inhibitor that disrupts the MDM2/p53 interaction) and NSC207895 (a small molecule that inhibits the MDMX promoter activity) co-treatment has a profound inhibitory effect on androgen-responsive prostate cancer LNCaP and 22RV1 cells that carry a wild-type copy of the *TP53* gene. This combinatorial inhibition not only activates p53, but also decreases the cellular levels of AR and its function. Furthermore, we demonstrate that co-expression of MDM2 and MDMX leads to stabilization of AR, and that MDMX modulates the MDM2-mediated AR ubiquitination. Therefore, combinatorial inhibition of MDM2 and MDMX may offer a novel strategy for prostate cancer therapy by promoting the p53 function and repressing AR function.

## RESULTS

### MDM2 and MDMX are co-amplified in CRPC datasets

**Figure 1:**
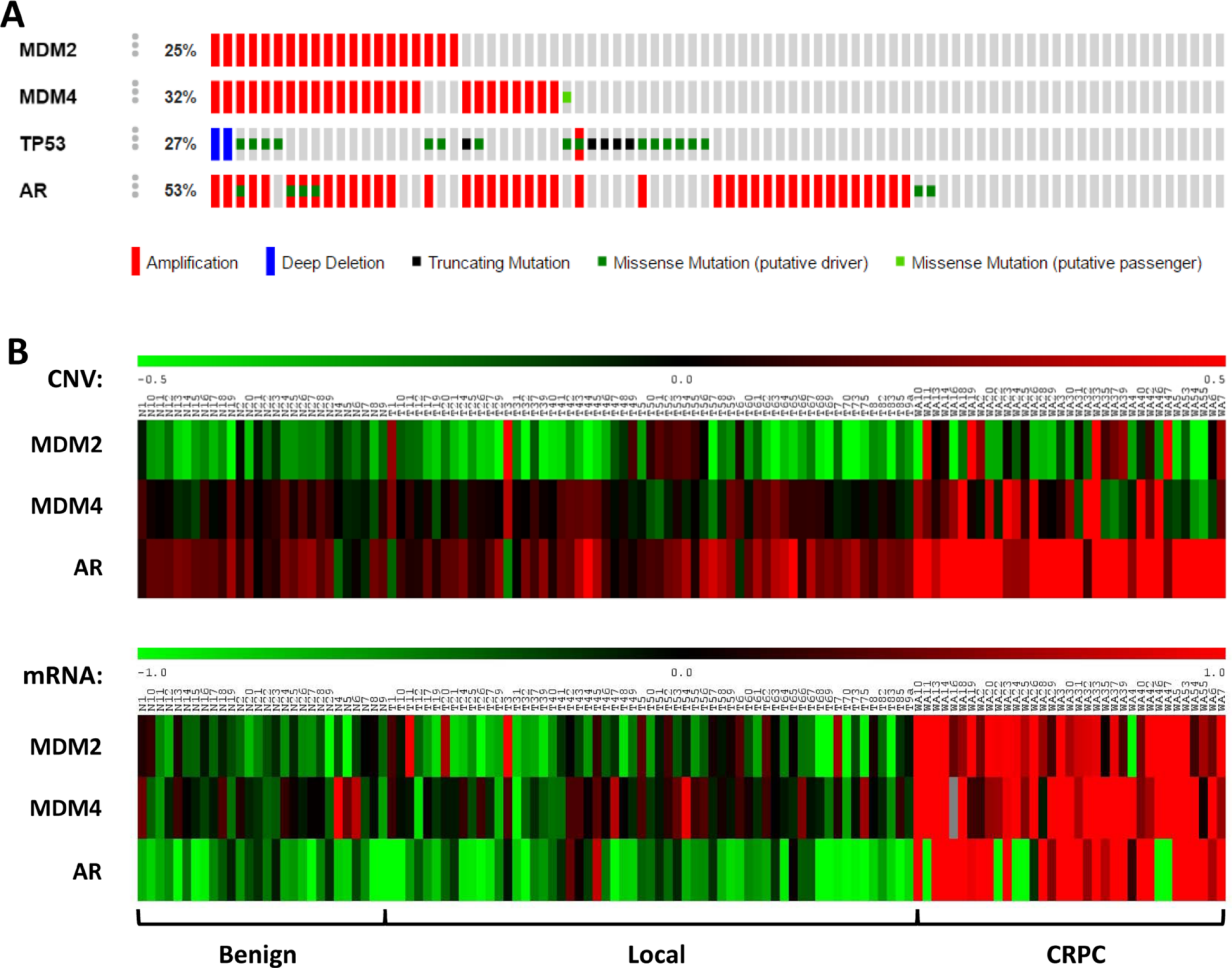
MDM2 and MDMX are co-amplified in CRPC (**A**) Prostate adenocarcinoma dataset (Trento/Cornell/Broad 2016; 114 samples) was analyzed using *cBioPortal*. MDM2 and MDMX are amplified in 25% and 32% of 114 CRPC cases, respectively. 85% (17/20) of samples with MDM2 amplification also exhibit MDMX amplification. Androgen receptor (AR) is amplified in about 50% of the samples. MDM2 and MDMX amplification is more frequent in samples with a wild-type *TP53* allele, consistent with their negative regulation of p53. (**B**) Copy number and gene expression analysis of a matched cohort of benign prostate tissues, localized prostate cancers, and metastatic CRPC samples (GSE35988). Copy number (aCGH) and gene expression data from a GEO publically available dataset (GSE35988) were obtained and analyzed by *GEO2R* to determine copy number and gene expression changes of MDM2, MDMX, and AR on a matched cohort of benign prostate tissues (*N* = 28), localized prostate cancers (*N* = 59), and metastatic CRPC samples (*N* = 35). The heatmap was generated using *MeV* software.

The p53 pathway is impaired in almost all human cancers, and about 50% of cancer cells sustain mutations in the *TP53* gene [35]. Although majority of the early-stage prostate cancer cells have wild-type *TP53* gene [36], recent studies have indicated that deregulation of p53 plays an important role in the advancement and metastatic potential of the disease [37–41]. In addition, overexpression of MDM2 has been observed in prostate carcinoma and associated with increased cell proliferation and tumor volume in prostate cancer, presumably by suppression of p53 function [42]. To investigate the role of p53 pathway in prostate cancer progression, we analyzed the prostate cancer genomic datasets in TCGA using *cBioPortal*. Our analysis of a CRPC dataset (Trento/Cornell/Broad 2016) revealed that MDMX is amplified in 32% of CRPC patients, compared to MDM2, which is amplified in 25% of CRPC patients (Figure 1A). Intriguingly, 85% of samples with MDM2 amplification co-exists with MDMX amplification. This is very unique, since overexpression of both genes is thought to be mutually exclusive in human cancer [28] (Supplementary Figure 1). Most of the MDM2 and/or MDMX amplifications exist in a wild-type p53 background, confirming their critical roles in p53 regulation. However, about 35% of MDM2/MDMX co-amplifications co-exist with p53 deletion or mutation, suggesting that they may also have a p53-independent oncogenic function in CRPC prostate cancer (Figure 1A). In addition, analyzing another publicly available CRPC dataset (GEO dataset number GSE35988) [43], we found that more MDM2 and MDMX co-amplification in metastatic CRPC patient samples comparing to benign or local tumor samples (Figure 1B, upper panel). Most strikingly, MDM2 and MDMX were co-overexpressed at mRNA level in majority of the CRPC samples (Figure 1B, lower panel). Together, our data revealed a unique co-amplification/overexpression of MDM2 and MDMX in CRPC.

### NSC/nutlin-3 co-treatment suppresses growth of prostate cancer cells

**Figure 2:**
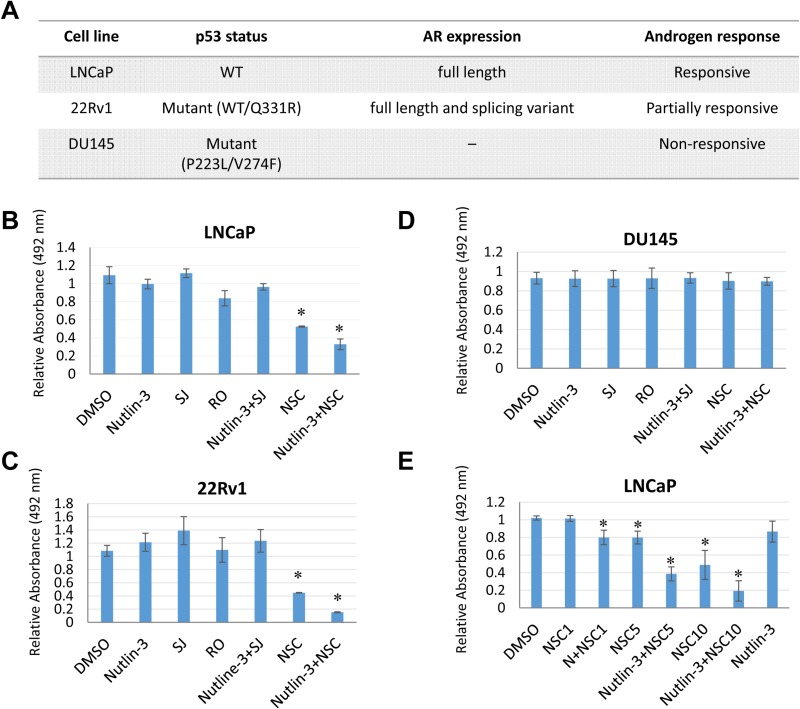
MDM2/MDMX inhibitors have various effect on the cell growth of prostate cancer cells (**A**) Genomic and expression phenotypes of prostate cancer cells used in this study. (**B**–**D**) Effect of MDM2/MDMX inhibitors on the growth of prostate cancer cells. LNCaP (B), 22Rv1 (C), and DU145 cells (D) were incubated 30 hours with nutlin-3 (5 µM), SJ (SJ-172550; 10 µM), RO (RO-5963; 10 µM), and NSC (NSC207895; 10 µM), alone or in combination, and cell proliferation was measured. The absorbance at 492 nm of DMSO treated cells was considered as 100% (as 1 in the bar chart). The results are expressed as means±s.d. of three independent experiments, each run in triplicates. The asterisk indicates statistical significance (*P* value < 0.05). (**E**) Dose-dependent inhibitory effect of nutlin-3 and NSC207895 on cell proliferation of LNCaP cells. LNCaP cells were incubated 30 hours with increasing dosage of NSC (NSC1, 1 µM; NSC5, 5 µM; NSC10, 10 µM) alone or in combination with nutlin-3 (5 µM), and cell proliferation was measured.

**Figure 3:**
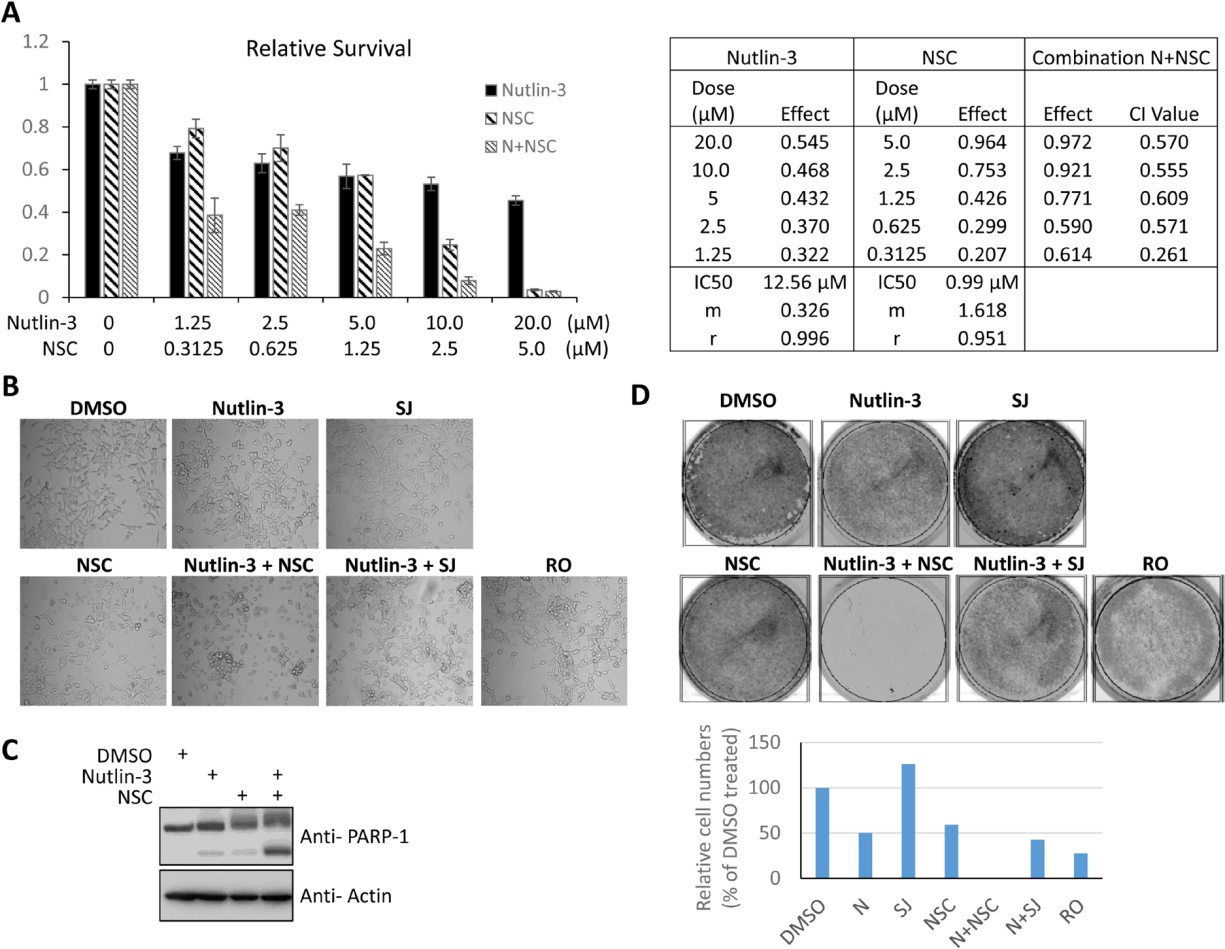
NSC/nutlin-3 co-treatment inhibits the cell growth of prostate cancer cells (**A**) NSC and nutlin-3 have a synergistic inhibitory effect on cell growth of LNCaP cells. LNCaP cells were treated with increasing concentration of nutlin-3 (0, 1.25, 2.5, 5.0, 10.0, or 20.0 µM), NSC207895 (0, 0.3125, 0.625, 1.25, 2.5, or 5.0 µM) or in combination as indicated for 30 hours and cell viability was measured. The absorbance at 492 nm of DMSO-treated cells was considered as 100% of survival (as 1 in the bar chart). The effects of drug treatment as fraction of DMSO-treated control cells were calculated. The synergistic analysis for the combination treatment was carried out using *CompuSyn* software as described in Materials and Methods. CI < 0.9 indicated a synergistic effect of NSC/nutlin-3 combination. (**B**) NSC/nutlin-3 co-treatment induces cell death in LNCaP cells. LNCaP cells were treated with MDM2/MDMX inhibitors alone (nutlin-3, 5 µM; SJ-172550, 10 µM; NSC207895, 10 µM; RO-5963, 10 µM) or in combination as indicated for 30 hours. The cells were examined for morphological change under a phase-contrast microscope (Nikon). (**C**) NSC/nutlin-3 co-treatment induces PARP-1 cleavage in LNCaP cells. LNCaP cells were treated with MDM2/MDMX inhibitors alone (nutlin-3, 5 µM; SJ-172550, 10 µM; NSC207895, 10 µM; RO-5963, 10 µM) or in combination as indicated for 30 hours. The whole cell lysates were analyzed using anti-PARP-1 and anti-actin antibodies. (**D**) NSC/nutlin-3 co-treatment suppresses growth of LNCaP cells in a colony formation assay. 1 × 10^5^ LNCaP cells were plated in 6-well plate and then treated with MDM2/MDMX inhibitors alone (nutlin-3, 5 µM; SJ-172550, 10 µM; NSC207895, 10 µM; RO-5963, 10 µM) or in combination as indicated. The medium was replaced every other days. Five days after treatment the cells were fixed and stained with crystal violet (0.05% in 20% of ethanol) to visualize the cell viability. The crystal violet stained cells were quantified using *LI-COR Odyssey CLx* imaging system. The absorbance of DMSO treated cells was considered as 100%. N: nutlin-3, SJ: SJ-172550; RO: RO-5963; NSC: NSC207895.

To test the hypothesis that combined inhibition of MDM2 and MDMX suppresses cell growth of prostate cancer cells, we examine the effect of various MDM2/MDMX inhibitors (Supplementary Figure 2) on cell proliferation of three different prostate cancer cell lines (Figure 2A): LNCaP cells are responsive to androgen and contain the wild-type p53 gene. 22Rv1 cells are partially responsive to androgen and contain one wild-type copy of p53 and one mutated copy of p53. DU145 cells are unresponsive to androgen and contain a mutant p53 [44]. Upon treatment with 5 nM nutlin-3 [45] (an MDM2 inhibitor), 20 µM SJ172550 [46] (SJ, an MDMX inhibitor), 10 µM RO5963 [47] (RO, a dual inhibitor of both MDM2 and MDMX), or a combination of 5 nM nutlin-3 and 20 µM SJ, none of the cells exhibited a growth inhibition (Figure 2B–2D). Intriguingly, NSC207895 (NSC), an MDMX inhibitor that blocks the MDMX promoter, thus inhibiting the MDMX expression [48], inhibited cell proliferation of both LNCaP (Figure 2B) and 22Rv1 cells (Figure 2C), but not DU145 cells (Figure 2D). The effect of NSC on cell proliferation was dose-dependent (Figure 2E). Moreover, co-treatment of nutlin-3 with NSC had a synergetic inhibitory effect on cell growth of LNCaP cells (Figures 3A). Cell growth inhibition was also observed in other p53 wild-type cell lines such as A549 (lung cancer) and U2OS (osteosarcoma) cells (Supplementary Figure 3B and 3C, respectively). The inhibition of cell growth by nutlin-3 and NSC co-treatment may mainly due to a cell death related mechanism. It was evidenced by the cell morphology changes (Figure 3B; more rounded up floating cells), FACS analysis (Supplementary Figure 3A; more subG1 species), as well as western blot analysis of PARP-1 (Figure 3C; more PARP-1 cleavage fragments). However, we couldn’t rule out the possibility that cell cycle arrest also contributed. Furthermore, in a colony formation assay, no viable cells were detected for LNCaP cells that were co-treated with nutlin-3 and NSC (Figure 3D). Less colony formation was also observed for cells that were treated in nutlin-3, nutlin-3 plus SJ, or RO. This is consistent with the previous reports that activation of p53 by MDM2 and MDMX inhibitors inhibit cell growth [45–47].

**Figure 4:**
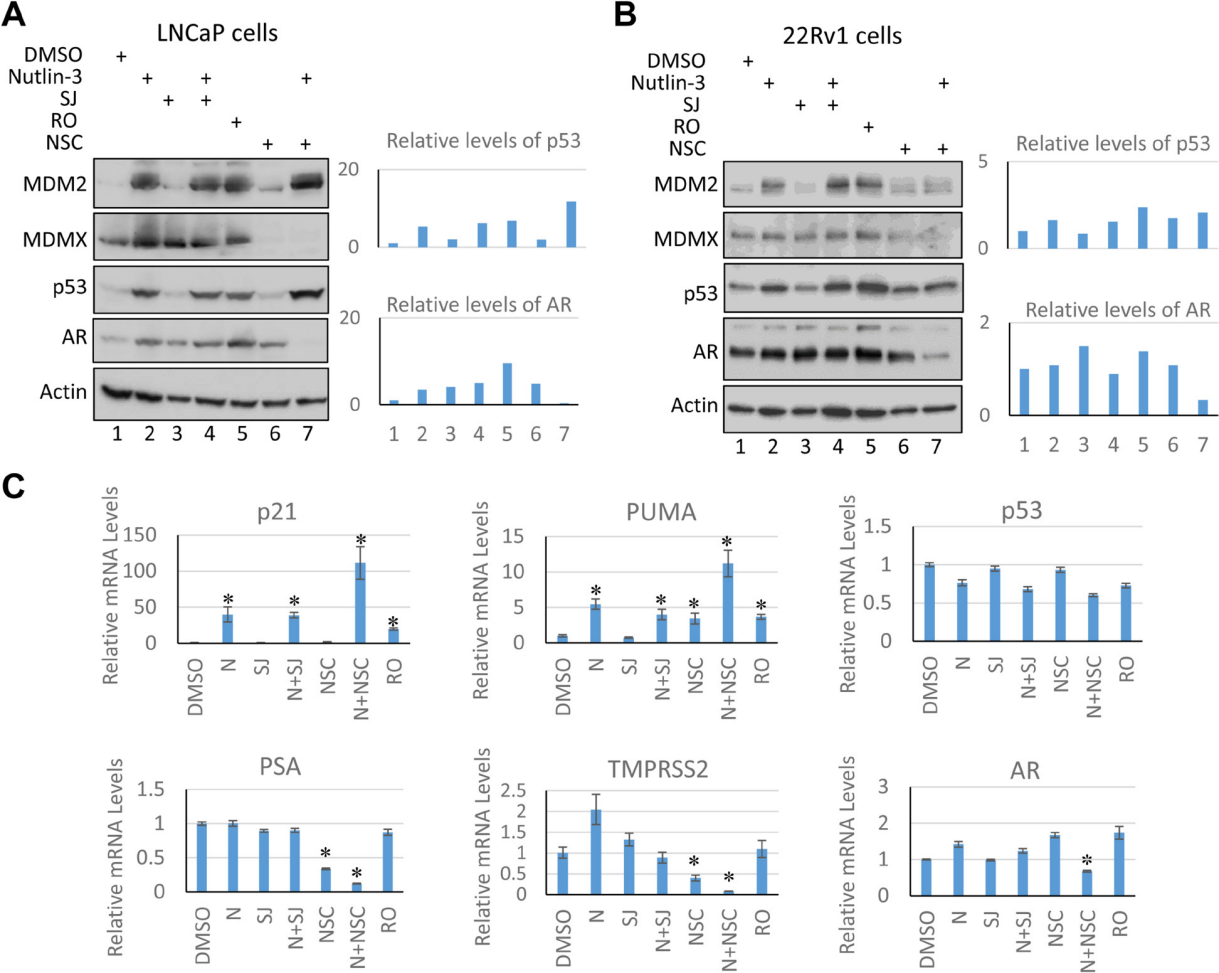
NSC/nutlin-3 co-treatment reduces AR cellular levels Immunoblotting analysis of LNCaP (**A**) and 22Rv1 (**B**) cells incubated 30 hours with nutlin-3 (5 µM), SJ (SJ-172550; 10 µM), RO (RO-5963, 10 µM), and NSC (NSC207895, 10 µM), alone and in combination. The whole cell lysates were analyzed using anti-AR, anti-MDM2, anti-MDMX, anti-p53, and anti-actin antibodies. Quantification the intensities of p53 and AR blots were carried out using *Image J* software. Note that there are two forms of AR in 22Rv1 cells: full-length AR and an alternative spliced form of AR that is smaller in size but expressed more. (**C**) Quantitative RT-PCR analysis of PSA, TMPRSS2, p21, PUMA, AR, and p53 mRNA levels in LNCaP cells incubated with MDM2/MDMX inhibitors as described in (A). The asterisk indicates statistical significance (*P* value < 0.05). N: nutlin-3, SJ: SJ-172550; RO: RO-5963; NSC: NSC207895.

Nutlin-3, which disrupts the interaction between MDM2 and p53, SJ, which disrupts the interaction between MDMX and p53, and RO, which disrupts the interaction between MDM2/MDMX and p53, all release p53 from the inhibitory effect of MDM2/MDMX, thus stabilizing and activating p53, and leading to cell cycle arrest or cell death [45–47]. Indeed, we found that both nutlin-3 and the dual inhibitor RO were able to stabilize p53 and induce the expression of MDM2, a p53 transcriptional target, in LNCaP cells (Figure 4A). On the other hand, MDMX inhibitors SJ and NSC did not stabilize p53 under the conditions used, which is consistent with the notion that MDM2, but not MDMX, is the major regulator of p53 stability [49]. Co-treatment using nutlin-3 with SJ or NSC led to p53 stabilization and activation. Consistent with the NSC inhibitory function in regulating MDMX expression [47], the MDMX protein levels were substantially decreased in LNCaP cells treated with NSC (Figure 4A). Similar results were obtained in 22Rv1 cells, suggesting that the wild-type copy of *TP53* in 22Rv1 cells retains its function (Figure 4B). Quantitative RT-PCR analysis revealed that mRNA levels of p53 targets p21 and PUMA were induced upon p53 induction in LNCaP cells (Figure 4C). Furthermore, RNA-seq analysis comparing LNCaP cells treated with nutlin-3, NSC, or in combination showed that p53 targets were induced to higher level in nutlin-3 and NSC co-treated cells comparing to those in cells treated with nutlin-3 only (Supplementary Figure 4A). This is in line with the synergistic effect of the co-treatment on cell growth inhibition. Together, our results demonstrate that co-inhibition of MDM2 (by nutlin-3) and MDMX (by NSC) has a profound inhibitory effect on cell growth of the wild-type p53 containing prostate cancer cells.

### NSC/nutlin-3 co-treatment reduces AR cellular levels

As shown in Figure 1, about half of all tumor samples in the Trento/Cornell/Broad dataset and majority of tumor samples in GSE35988 dataset harbor AR amplification and/or AR mutations, leading to promiscuity. Both MDM2 and p53 have been shown to inhibit the AR expression [50–53]. Moreover, it has been reported that nutlin-3 reduces AR cellular levels in androgen-depleted LNCaP cells, and that combinational treatment of nutlin-3 with androgen depletion sensitizes LNCaP cells to apoptosis [32]. To examine the effect of MDM2/MDMX inhibition on the AR expression, we analyzed the cellular levels of AR in LNCaP (Figure 4A) and 22Rv1 (Figure 4B) cells treated with the MDM2/MDMX inhibitors. The cellular levels of AR in LNCaP and 22Rv1 cells were unchanged or increased in cells treated with the individual MDM2/MDMX inhibitors and with the combination of nutlin-3 and SJ. Importantly, the combination of nutlin-3 and NSC substantially decreased the cellular levels of AR in both cell types (Figures 4A and 4B). Interestingly, a smaller sized alternative spliced form of AR was the dominant form in 22Rv1 cells, as previously shown [54]. Moreover, quantitative RT-PCR analysis revealed that mRNA levels of prostate specific antigen (PSA), an AR target that is often used as a measure of androgen receptor signaling and marker of prostate cancer progression [55], significantly decreased following NSC treatment, especially in combination with nutlin-3 (Figure 4C). Similar reduction was observed for another well-known AR target, TMPRSS2 (Figure 4C). Moreover, Gene Set Enrichment Analysis (GSEA) of RNA-seq data using a list of 83 androgen receptor signaling target genes (Androgen Receptor Signaling Targets PCR Array; Qiagen) revealed that AR signaling was down-regulated in cells that were co-treated with nutlin-3 and NSC (Supplementary Figure 4B). A reproducible small reduction on mRNA levels of AR was also observed in cells that were co-treated with nutlin-3 and NSC by quantitative RT-PCR analysis (Figure 4C). In contrast, NSC and NSC with nutlin-3 had a limited effect on the mRNA levels of p53 (Figure 4C).

### Co-expression of MDM2 and MDMX stabilizes AR

**Figure 5:**
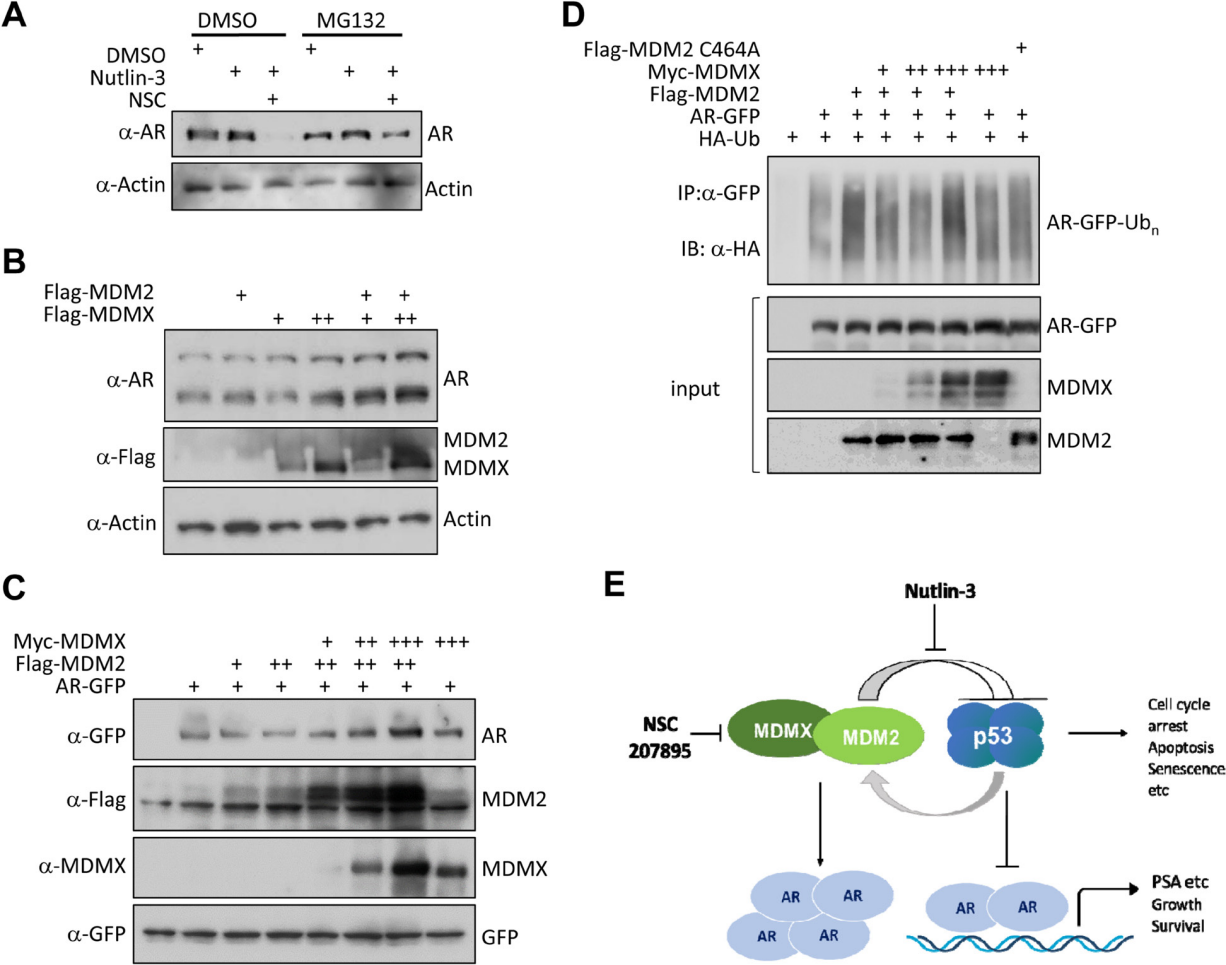
Co-expression of MDM2 and MDMX stabilizes AR (**A**) MG132 rescued the levels of AR in cells co-treated with NSC/nutlin-3. LNCaP cells were incubated 24 hours with DMSO, nutlin-3 (5 µM), or combination of nutlin-3 (5 µM) and NSC (NSC207895, 10 µM), and then treated with or without MG132 (20 µM) for 6 hours. The whole cell lysates were analyzed by immunoblotting using anti-AR and anti-actin antibodies. (**B**) Co-expression of MDM2 and MDMX stabilizes endogenous AR in 22Rv1 cells. 22Rv1 cells were transfected with Flag-MDM2 (1.5 µg) or Flag-MDMX (0.4 or 0.8 µg) alone and in combination, as indicated. Twenty-four hours after transfection, total cell lysates were prepared, and analyzed by immunoblotting with anti-AR, anti-Flag, and anti-actin antibodies. Note that there are two forms of AR in 22Rv1 cells: full-length AR and an alternative spliced form of AR that is smaller in size but expressed more. (**C**) Co-expression of MDM2 and MDMX stabilizes ectopically expressed AR-GFP fusion protein. HEK293 cells were transfected with AR-GFP (0.3 µg), Flag-MDM2 (1.2 µg), or Myc-MDMX (0.2, 0.4 or 0.8 µg) alone and in combination, as indicated. Twenty-four hours after transfection, total cell lysates were prepared, and analyzed by immunoblotting with anti-GFP, anti-MDM2, and anti-MDMX antibodies. A co-expressed GFP construct was added in each case to control for transfection efficiency and loading. (**D**) MDMX inhibits MDM2-mediated AR ubiquitination. HEK293 cells were transfected with the indicated combinations of AR-GFP (0.3 µg), Flag-MDM2 (1.2 µg), MDM2 C464A (0.6 µg), or Myc-MDMX (0.2, 0.4 or 0.8 µg) along with an HA-ubiquitin (HA-Ub; 1.2 μg) plasmid as indicated. The cells were treated with MG132 (20 μM) for 6 h before harvesting. Whole-cell lysates were subjected to immunoprecipitation with anti-GFP antibody followed by immunoblotting with anti-HA antibody to detect ubiquitinated AR-GFP. 10% of input was analyzed by immunoblotting with anti-GFP, anti-MDM2, and anti-MDMX antibodies. (**E**) A proposed function of the MDM2/MDMX co-amplification in CRPC progression. MDM2 and MDMX uniquely co-amplify/co-overexpress in CRPC. Firstly, they inhibit p53 tumor suppression function. Secondly, they release the transcriptional repression effect of p53 on AR signaling. Thirdly, they stabilize AR to promote AR signaling. Together, they result in increased cell growth and survival of prostate cancer cells. Combinatorial inhibition of MDM2 and MDMX by nutlin-3 and NSC207895, respectively, activates p53 signaling while represses AR signaling, offering a compelling strategy for prostate cancer therapy.

As mRNA levels of AR were slightly but protein levels of AR were dramatically reduced by nutlin-3 and NSC co-treatment (Figure 4), we reasoned if AR stability was also regulated at post-transcriptional level. Indeed, as shown in Figure 5A, cellular levels of AR were partially rescued by treatment of MG132, a proteasome inhibitor. MDM2, an E3 ubiquitin ligase, is involved in Akt-mediated AR ubiquitination and degradation [51]. Under our experimental conditions, we found that only NSC/nutlin-3 co-treatment led to the reduced AR expression and activity, although various treatments resulted in the MDM2 induction (Figure 4). This result suggested that cellular levels of MDMX might modulate the ability of MDM2 to degrade AR. Since it has been shown that MDMX forms a heterodimer with MDM2 and regulates its E3 ligase activity [24–27], we tested whether MDMX regulates the AR stability through MDM2, by using ectopically expressed MDM2 and MDMX proteins. As shown in Figure 5B, co-expression of MDM2 and MDMX increased endogenous AR protein levels in 22Rv1 cells for both full-length and a smaller sized alternative spliced form of AR. Furthermore, co-expression of MDM2 and MDMX increased levels of co-transfected AR-GFP, a hybrid gene under the CMV promoter (Figure 5C), indicating that MDM2/MDMX co-expression increases the stability of AR in prostate cancer cells.

As AR has been shown to be an E3 ligase target of MDM2, we further carried out an *in vivo* ubiquitination assay to examine whether MDMX modulates the MDM2-mediated AR ubiquitination. As shown in Figure 5D, co-transfection with MDM2 but not a MDM2 E3 ligase defective mutant (C464A) greatly increased the AR-GFP ubiquitination. This is consistent with a previous report indicating that MDM2 mediates the AR ubiquitination [51]. Intriguingly, co-expression of MDMX inhibited MDM2-mediated AR ubiquitination at lower dosages but lost this inhibitory effect at a higher dosage (Figure 5D). Therefore, the ratio between MDM2 and MDMX seems to be important for MDM2-mediated AR ubiquitination. Together, our results indicate that MDMX regulates the AR stability in prostate cancer cells by modulating the MDM2 E3 ligase function towards AR.

## DISCUSSION

Since patients with CRPC have very limited treatment options, better therapeutic strategies are urgently needed. Although overexpression of MDM2 has been linked to advanced stages of prostate cancer, the role of MDMX in prostate cancer progression remains unclear. By analyzing TCGA datasets, we found that MDMX is amplified at a higher rate than MDM2 in CRPC. Interestingly, about 85% of MDM2 amplifications co-exist with MDMX amplification, a phenomenon rarely observed in other cancer types (Figure 1A and Supplementary Figure 1). We also observed that more CRPC samples harbored MDM2 and MDMX co-amplification/co-overexpression than benign or localized prostate tumor samples (Figure 1B). We anticipated that co-amplification/co-overexpression of MDM2 and MDMX, two crucial negative regulators of p53, provides a unique mechanism for CRPC progression, and combinatorial inhibition of MDM2 and MDMX offers a compelling strategy for CRPC therapy. We found that in addition to the well-known abilities of MDM2 and MDMX to inhibit the p53 function, MDM2 and MDMX co-expression stabilizes AR. Combinatorial inhibition of MDM2 and MDMX that not only activates p53 but also represses AR signaling led to more potent growth inhibition for prostate cancer cells comparing to treatment that only activates p53. Thus, our data suggest that MDM2 and MDMX co-amplification/co-overexpression modulates both p53 and AR signaling pathways, contributing to CRPC progression (Figure 5E).

Previous studies have indicated the crosstalk between p53 and AR signaling. Overexpression of p53 inhibits expression of androgen-dependent genes [50], while inhibition of p53 function diminishes AR-mediated signaling in prostate cancer cells [53]. Similarly, it has been observed that etoposide-induced p53 binds to the AR gene promoter to attenuate androgen signaling in prostate cancer LNCaP cells [52]. Moreover, genome-wide ChIP-sequence analysis has revealed that p53 suppression affects the AR specificity to chromatin binding and regulation of gene expression in prostate cancer cells [56]. About 70% of the MDM2/MDMX co-amplified CRPC tumor samples retain wild-type p53, suggesting that dysregulation of only one of the inhibitors is not sufficient to inactivate p53 for prostate cancer development. It is possible that in prostate cancer cells, p53 has cell-type specific function that is tightly controlled by both MDM2 and MDMX. Alternatively, a p53-independent function of MDM2 or MDMX needs to be compromised in addition to p53 inactivation for prostate cancer progression. Indeed, about 30% of the tumor samples with MDM2/MDMX co-amplification have p53 mutation or deletion.

Using a panel of MDM2/MDMX inhibitors, we examined the effect of the dual inhibition of p53-related MDM2 and MDMX functions on the cell growth of prostate cancer cells. When LNCaP and 22Rv1 cells, two prostate cancer cell lines carrying the wild-type copy of the *TP53* gene, were treated with the dual inhibitor RO, or with a combination of nutlin-3 and SJ, p53 was stabilized and activated, since both treatments disrupted the p53 interaction with MDM2 and MDMX. However, the cell growth of both cell types was not affected under the conditions used. It has been shown that nutlin-3 boosts the antitumor effect of androgen withdrawal for prostate cancer therapy [32]. It is believed that this effect is in part due to the inhibition of AR expression caused by p53 activation. However, under our experimental conditions using normal growth medium, we did not observe any changes in AR expression from nutlin-3 alone or in combination with SJ, although p53 was activated as expected. Therefore, androgen withdrawal may induce additional factors that facilitate the p53-mediated inhibition of AR expression. Further investigation will be needed to explore this possibility.

Interestingly, NSC treatment, especially in combination with nutlin-3, had a profound inhibitory effect on the growth of prostate cancer cells carrying the wild-type p53. NSC (a 4-nitrobenzofuroxan derivative, also named XI-006) was identified through a high-throughput drug screening for compounds that could mitigate the MDMX promoter activity. NSC represses the MDMX promoter activity, resulting in decreased MDMX mRNA and protein expression, and reduced cell viability in MDMX amplified breast cancer cells [48]. Furthermore, it has been shown that NSC induces p53-independent apoptosis in Ewing sarcoma [57]. We found that NSC treatment, especially in combination with nutlin-3, had a profound inhibitory effect on AR signaling as evidenced by quantitative RT-PCR and RNA-seq analysis (Figure 4C and Supplementary Figure 4). Therefore, reduced cellular levels of MDMX upon NSC treatment may augments the effect of nutlin-3 to activate p53. In addition, NSC may employ MDMX-dependent but p53-independent mechanism(s), in combination with its effect on nutlin-3 induced p53 activation, to inhibit cell growth and repress AR signaling. Further investigation will be required to examine whether MDMX protects the androgen target genes from p53-mediated transcriptional repression and whether MDMX promotes AR signaling independent of p53.

Intriguingly, we observed that co-treatment of NSC and nutlin-3 destabilizes AR and MG132 treatment allows the rescue of expression of AR, suggesting a post-transcriptional regulation of AR by NSC and nutlin-3 co-treatment. Furthermore, our results show that MDM2 and MDMX co-expression stabilizes both ectopically co-expressed AR and endogenous AR protein. Accumulation of the ectopically expressed AR protein under CMV promoter suggests that the stabilization of AR can happen at the post-transcriptional level. We have tried but failed to recapitulate the effect of NSC/nutlin-3 co-treatment on AR stability in LNCaP cells using siRNA targeting MDMX in combination with nutlin-3 treatment (data not shown). We speculate the discrepancy between MDMX knock-down and NSC treatment may due to the fact that NSC treatment is more effective in reducing cellular levels of MDMX in comparison to MDMX siRNA treatment. As NSC structurally clustered with known DNA-damaging agents such as camptothecin [58], it may induce MDMX degradation more efficiently comparing to siRNA knockdown. In addition, it may induce AR phosphorylation therefore make it more vulnerable for MDM2-mediated degradation. Note that MDM2 has been shown to modulate AR protein levels by targeting AR for ubiquitination and degradation in Akt-dependent manner [51]. Under our experimental conditions, we did not observe a reduction in cellular levels of neither the ectopically expressed nor the endogenous AR upon MDM2 overexpression, although MDM2 did mediate AR ubiquitination in the *in vivo* ubiquitination assay (Figure 5). Co-expression of MDMX modulates MDM2-mediated AR ubiquitination, although it seems that their effect on AR ubiquitination is uncoupled from their ability to stabilize AR (Figure 5C and 5D). Nevertheless, MDM2 and MDMX are involved in AR ubiquitination and degradation. Their ability to regulate AR stability may play a vital role in CRPC progression. As majority (∼85%) of the CRPC samples with MDM2/MDMX co-amplification contain AR amplification regardless of p53 status, the ability of combinatorial inhibition of MDM2 and MDMX by nutlin-3 and NSC to target AR provides a viable strategy for CRPC therapy.

## MATERIALS AND METHODS

### Cell culture and plasmids

LNCaP cells were grown in RPMI medium containing 10% fetal bovine serum (FBS) at 37°C. 22Rv1, DU145, and HEK293 cells were grown in Dulbecco modified Eagle medium containing 10% FBS at 37°C. Construction of Flag-MDM2 and HA-Ubiquitin was described previously [59, 60]. pEGFP-C1-AR was a gift from Michael Mancini (Addgene plasmid # 28235). Flag-MDMX was constructed by subcloning MDMX fragment from Myc-MDMX construct [59] into Flag-MDM2 construct by replacing the MDM2 sequence.

### Antibodies and reagents

Commercially obtained antibodies were used to detect the following proteins or epitopes: androgen receptor (mouse monoclonal antibody; 441, Santa Cruz Biotechnology, USA), MDMX (rabbit polyclonal antibody; Bethyl), actin (mouse monoclonal antibody; C-4, Santa Cruz Biotechnology), Flag (mouse monoclonal antibody; M2, Sigma), HA (mouse monoclonal antibody; HA.11, Covance), and GFP (mouse monoclonal antibody; B-2, Santa Cruz Biotechnology). Mouse monoclonal antibodies against human p53 (DO-1) and MDM2 (3G5, 4B11, 5B10) were used as supernatants from hybridoma cultures. Inhibitors used in this study were as follows: nutlin-3 (5 μM; Sigma), SJ172550 (20 μM; Calbiochem), RO5963 (10 μM; Calbiochem), NSC207895 (1, 5, or 10 μM; Calbiochem), and MG132 (20 μM; Calbiochem).

### Transfection and Western blot analysis

Transfections in HEK 293 were performed using PEI (Polysciences; cat# 23966-2) with 1:3 ratio of DNA:PEI. In all transfection experiments, balancing amounts of empty vector (pcDNA3 plasmid) were added to ensure equal amounts of total DNA used for transfecting cells. Total cell lysates were prepared and separated on SDS polyacrylamide gels, transferred to nitrocellulose and immunoblotted with the indicated antibodies as described previously [61].

### Cell proliferation assay

Cell proliferation was measured by OD 492 nm using the CellTiter 96^®^ AQueous One Solution Cell Proliferation Assay (MTS, Promega). Approximately 3000 cells per well were plated in 96-well plates in 150 μl of complete medium (RPMI for LNCaP, and DMEM for 22Rv1 or DU145 cells) and allowed to attach for 20 hours under normal culture conditions. The cells in triplicates were treated with MDM2 or MDMX inhibitor alone or in combination, and incubated for 30 hours under normal culture conditions. Medium was then removed and replaced with 100 μl of fresh complete medium and 10 μl CellTiter 96^®^ AQueous One Solution, and incubated for 2 hours at 37°C. The plates were read on an Emax Plus Microplate Reader (Molecular Devices) at a wavelength of 492 nm. The results are expressed as means ± s.d. of three independent experiments.

For synergistic analysis, LNCaP cells were treated with increasing dosages of nutlin-3 (0, 1.25, 2.5, 5.0, 10.0, or 20.0 µM), NSC207895 (0, 0.3125, 0.625, 1.25, 2.5, or 5.0 µM) or in combination as indicated for 30 hours and cell viability was measured. The absorbance at 492 nm of DMSO-treated cells was considered as 100% of survival (as 1 in the bar chart). The effects of drug treatment as fraction of DMSO-treated control cells were calculated. The dose-response values IC50 (dose required for median effect), m (slope signifying the shape of the dose–response curve) and r (linear correlation coefficient; r = 1indicates perfect fit) for nutlin-3 and NSC were derived using Compusyn software (http://www.combosyn.com; [62]). Based on these values, the CI was derived for drug combination, reflecting the extent of synergy or antagonism for two drugs. CI < 0.9, synergy; 0.9 < CI < 1.1, additive effect; CI > 1.1, antagonism.

### Colony formation assay

1 × 10^5^ LNCaP cells were plated in 6-well plate and then treated with MDM2/MDMX inhibitors alone or in combination as indicated. The medium was replaced every other days. Five days after treatment the cells were fixed and stained with crystal violet (0.05% in 20% of ethanol) to visualize the cell viability. The crystal violet stained cells were scanned using LI-COR Odyssey CLx imaging system. The intensity of crystal violet signal in each well was quantified to represent the cell numbers in the well. The absorbance of DMSO treated cells was considered as 100%.

### *In vivo* ubiquitination assay

HEK293 cells were transfected with the indicated combinations of plasmids. Twenty-four hours after transfection, cells were treated with MG132 (20 μM) for 6 hours and then harvested. Total cell lysates were prepared and equal amounts of cleared cell lysates were subjected to anti-GFP (B2, Santa Cruz) immunoprecipitation followed by western blotting with the monoclonal anti-HA antibody (Covance).

### RNA extraction and quantitative RT-PCR analysis

LNCaP cells were plated in 6-well plates and treated with indicated combinations of MDM2 and MDMX inhibitors. Thirty hours after treatment, cells were harvested. RNA was extracted using a QIAGEN RNeasy mini-kit, and cDNA was synthesized with the QuantiTect reverse transcription kit (QIAGEN). Samples were analyzed by quantitative real-time PCR on a Bio-Rad CFX 96 using SYBR Green (Quantabio). RNA expression was normalized to RPL32 mRNA expression. Relative levels were calculated by the comparative *Ct* method (ΔΔC_*T*_ method). The results are expressed as means ± s.d. of four experiments. Primer sequences are: PSA (F: 5′-AGGCCTTCCCTGTACACCAA-3′; R: 5′- GTCTTGGCCTGGTCATTTCC-3′), TMPRSS2 (F: 5′-CTG GTG GCT GAT AGG GGA TA-3′; R: 5′-GGA CAA GGG GTT AGG GAG AG-3′), AR (F: 5′-CTGGACACGACAACAACCAG-3′; R: 5′- CAGATCAGGGGCGAAGTAGA-3′), p21 (F: 5′- GGCGGCAGACCAGCATGACAGATT-3′; R: 5′- GCAGGGGGCGGCCAGGGTAT-3′), PUMA (F: 5′-CCTGGAGGGTCCTGTACAATCT-3′; R: 5′-GCACCT-AATTGGGCTCCATCT-3′), L32 (F: 5′-TTCCTGGTCCACAATGTCAAG-3′; R: 5′- TGTGAGCGATCTCAGCAC-3′), and p53 (QIAGEN, cat#PPH00213F).

### Copy number and gene expression analysis

Prostate adenocarcinoma dataset (Trento/Cornell/Broad 2016; 114 samples; [63]) was analyzed using *cBioPortal*. Copy number (aCGH) and gene expression data from a GEO publically available dataset (GSE35988; [43]) were obtained and analyzed by GEO2R to determine copy number and gene expression changes of MDM2, MDMX, and AR on a matched cohort of benign prostate tissues (*N* = 28), localized prostate cancers (*N* = 59), and metastatic CRPC samples (*N* = 35). The heatmap was generated using MeV software (http://mev.tm4.org).

## SUPPLEMENTARY MATERIALS FIGURES


