# Supplementary material for: Activation of p53 and destabilization of androgen receptor by combinatorial inhibition of MDM2 and MDMX in prostate cancer cells
Source: Oncotarget. 2017 Dec 15;9(5):6270–81. doi: 10.18632/oncotarget.23569 (PMC5814211; doi:10.18632/oncotarget.23569)
Supplement: Supplementary file 1 [file oncotarget-09-6270-s001.pdf]

Activation of p53 and destabilization of androgen receptor by combinatorial inhibition of MDM2 and MDMX in prostate cancer cells

SUPPLEMENTARY MATERIALS

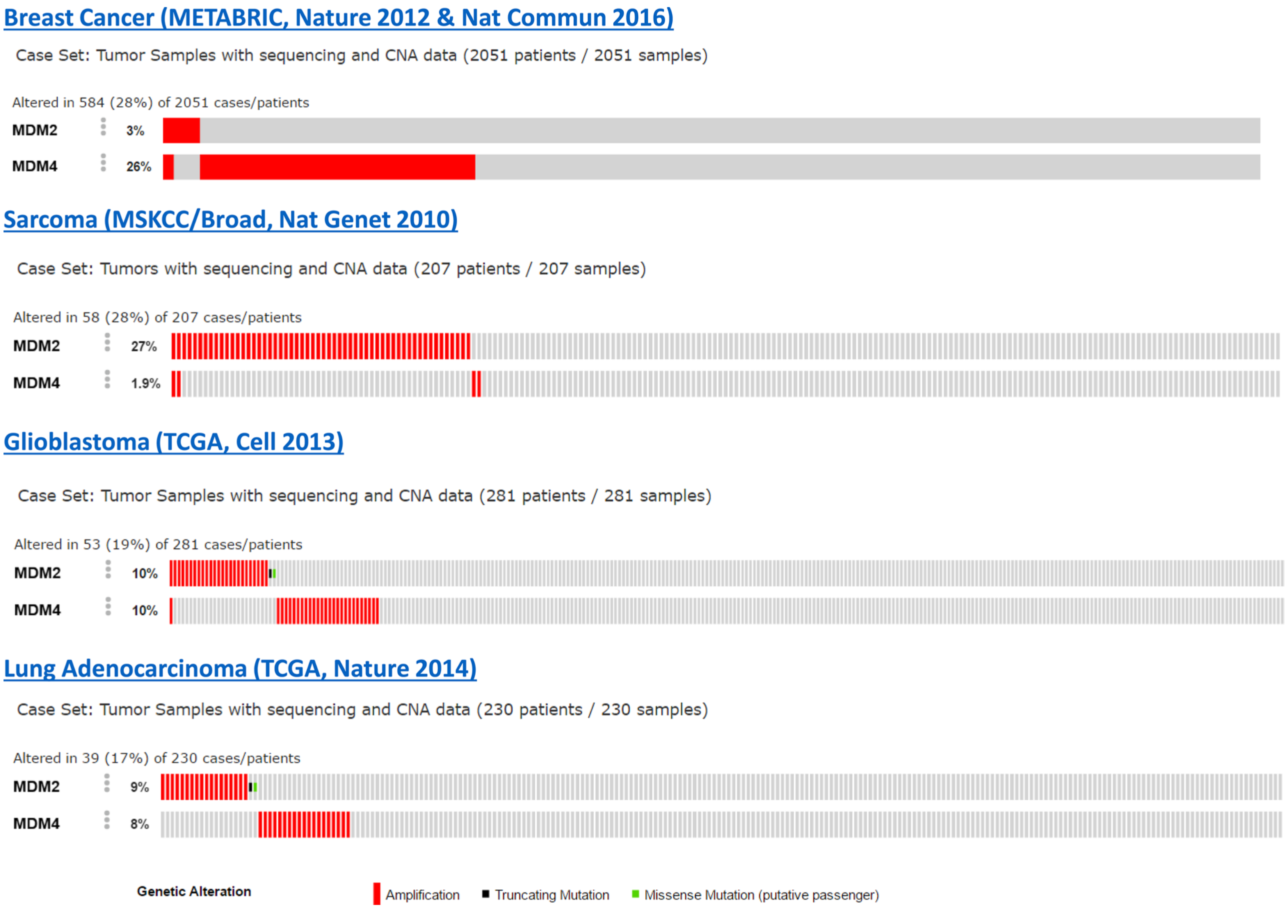

Supplementary Figure 1: MDM2 and MDMX are amplified and mutually exclusive in several human cancers. Breast cancer (METABRIC, Nature 2012 & Nat Commun 2016 [1, 2]; 2051 samples), sarcoma (MSKCC/Broad, Nat Genet 2010 [3]; 207 samples), glioblastoma (TCGA, Cell 2013 [4]; 281 samples), and lung adenocarcinoma (TCGA, Nature 2014 [5]; 230 samples) datasets were analyzed using *cBioPortal*. MDMX is preferentially amplified in breast cancer (MDMX:MDM2 = 26:3), while MDM2 is preferentially amplified in sarcoma (MDMX:MDM2 = 1.9:27). In glioblastoma and lung adenocarcinoma samples, both MDM2 and MDMX are amplified at similar frequency, but mutually exclusive in each patient sample.

Targeting MDM2/MDMX-p53 interaction

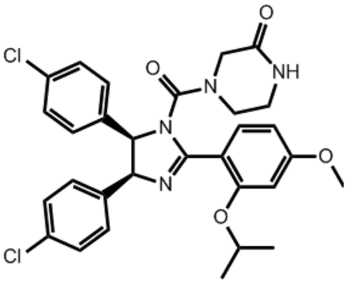

Nutlin-3a

MDM2-p53

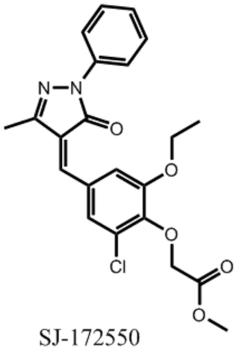

SJ-172550

MDMX-p53

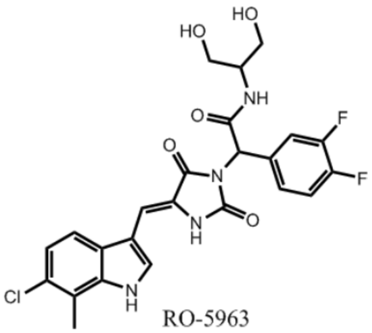

RO-5963

MDM2-p53 &
MDMX-p53

Targeting MDMX promoter

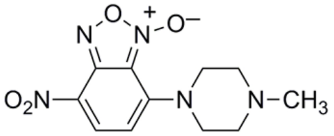

NSC207895

Supplementary Figure 2: Molecular structure of MDM2 and MDMX inhibitors used in this study.

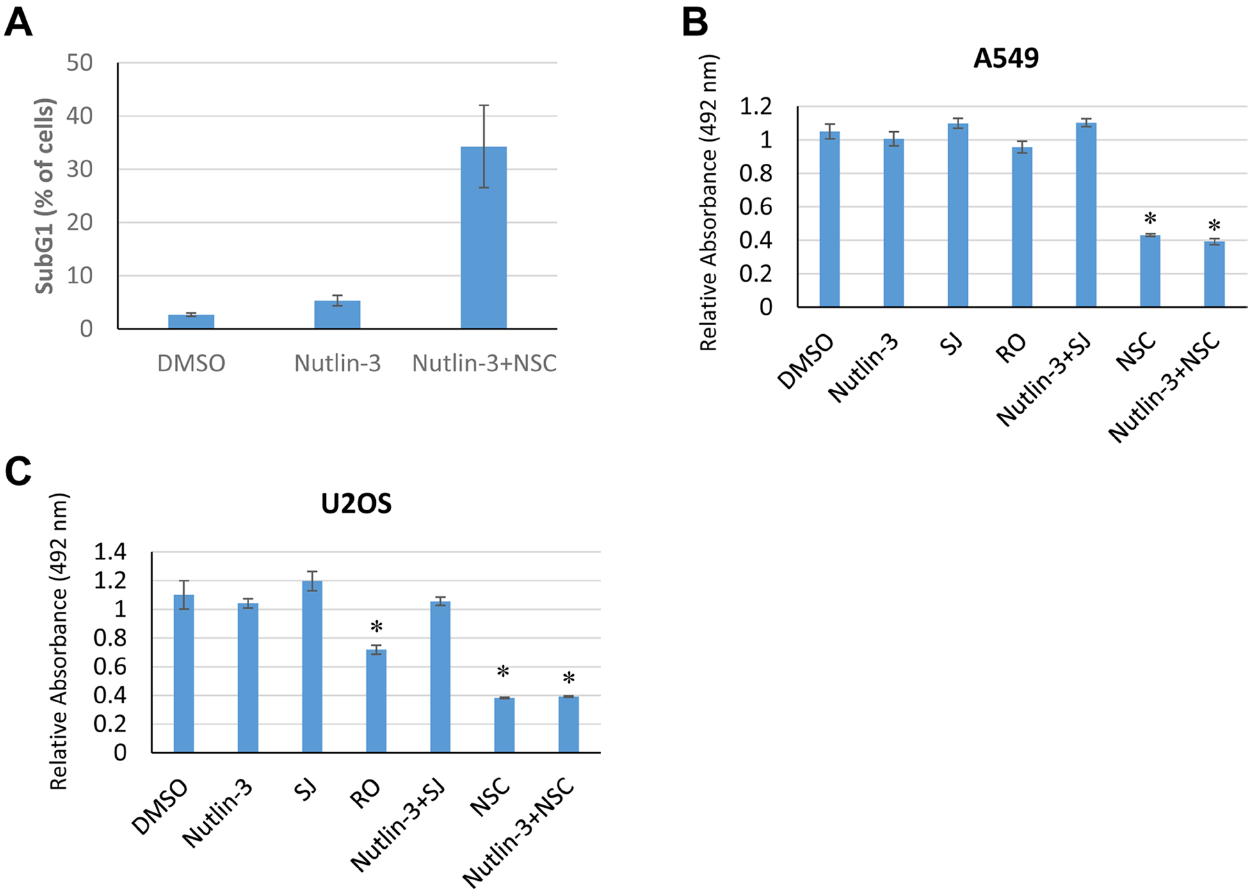

Supplementary Figure 3: NSC/nutlin-3 co-treatment induces cell death and inhibits cell growth. (**A**) NSC/nutlin-3 co-treatment induces cell death in LNCaP cells. LNCaP cells were treated with nutlin-3 and NSC for 30 hours and then were harvested by trypsinization, fixed, and resuspended in phosphate-buffered saline solution containing RNase (100 µg/ml) and propidium iodide (60 µg/ml, Sigma). Stained cells were analyzed in a fluorescence-activated cell sorter (Guava® easyCyte Flow Cytometers, Millipore). The percentage of subG1 cells was quantified using *InCyte* software. (**B** and **C**) NSC/nutlin-3 co-treatment inhibits cell growth. A549 cells (B) and U2OS cells (C) were incubated 30 hours with nutlin-3 (5 µM), SJ (SJ-172550; 10 µM), RO (RO-5963; 10 µM), and NSC (NSC207895; 10 µM), alone or in combination, and cell proliferation was measured. The absorbance at 492 nm of DMSO treated cells was considered as 100% (as 1 in the bar chart). The results are expressed as means±s.d. of three independent experiments, each run in triplicates. The asterisk indicates statistical significance (P value < 0.05).

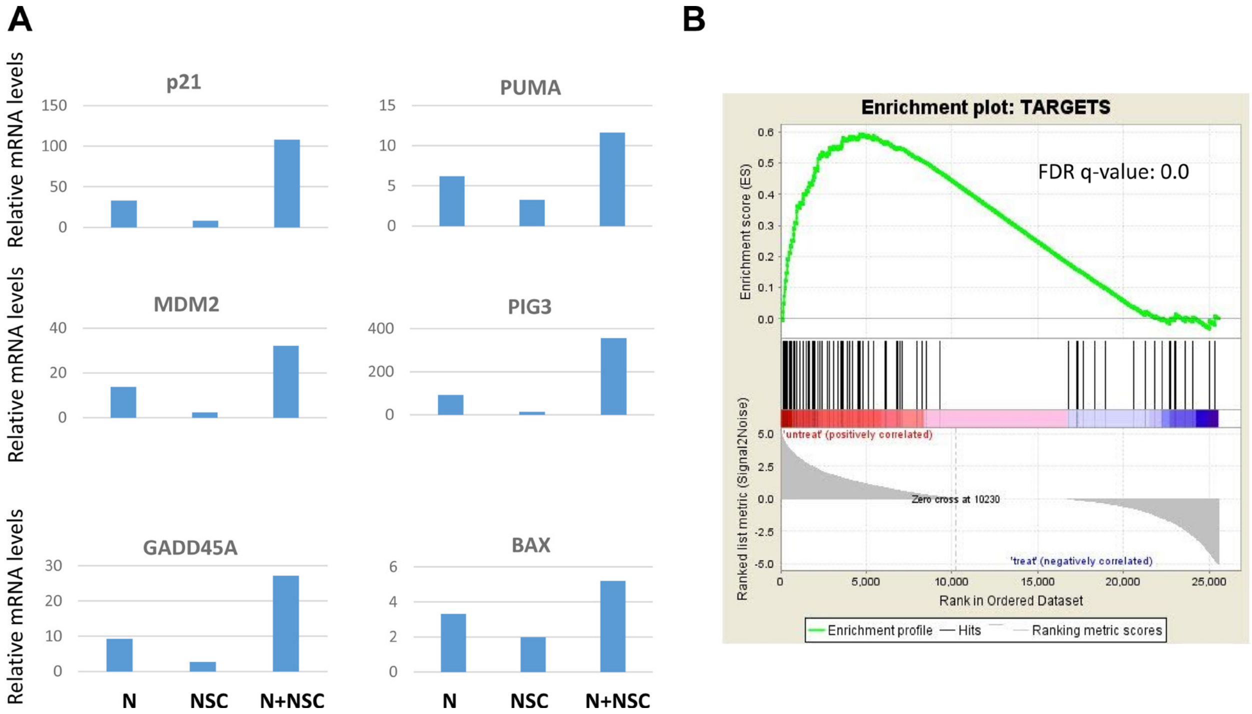

Supplementary Figure 4: NSC/nutlin-3 co-treatment activates p53 signaling pathway and represses AR signaling pathway. (**A**) RNA-seq analysis confirms the effect of NSC/nutlin-3 co-treatment on p53 activation. LNCaP cells were treated with DMSO, nutlin-3 (5 µM), NSC (NSC207895; 10 µM), or combination of nutlin-3 and NSC for 30 hours. Total RNAs were extracted and subjected to Bioanalyzer for quality control followed by RNA-seq analysis (Illumina 4000; 30M reads; Columbia genome center). Normalized expression levels (FPKM) of known p53 targets p21, PUMA, MDM2, PIG3, GADD45a, and BAX were analyzed and relative expression levels were calculated in comparison to DMSO-treated sample. (**B**) Expression of AR signaling targets were enriched in DMSO-treated but not NSC/nutlin-3 co-treated LNCaP cells. A list of 83 androgen receptor signaling target genes (Androgen Receptor Signaling Targets PCR Array; Qiagen; supplemental excel file) was analyzed using Gene Set Enrichment Analysis (http://software.broadinstitute.org/gsea/index.jsp; GSEA) to compare their expression levels in DMSO-treated or NSC/nutlin-3 co-treated RNA-seq dataset. Majority of these genes were found to be enriched in DMSO-treated samples, suggesting their expression were repressed in NSC/nutlin-3 co-treated sample.

REFERENCES

1. Curtis C, Shah SP, Chin SF, Turashvili G, Rueda OM, Dunning MJ, Speed D, Lynch AG, Samarajiwa S, Yuan Y, Graf S, Ha G, Haffari G, et al. The genomic and transcriptomic architecture of 2,000 breast tumours reveals novel subgroups. Nature. 2012; 486:346–352.
2. Pereira B, Chin SF, Rueda OM, Vollan HK, Provenzano E, Bardwell HA, Pugh M, Jones L, Russell R, Sammut SJ, Tsui DW, Liu B, Dawson SJ, et al. The somatic mutation profiles of 2,433 breast cancers refines their genomic and transcriptomic landscapes. Nat Commun. 2016; 7:11479.
3. Barretina J, Taylor BS, Banerji S, Ramos AH, Lagos-Quintana M, Decarolis PL, Shah K, Socci ND, Weir BA, Ho A, Chiang DY, Reva B, Mermel CH, et al. Subtype-specific genomic alterations define new targets for soft-tissue sarcoma therapy. Nat Genet. 2010; 42:715–721.
4. Brennan CW, Verhaak RG, McKenna A, Campos B, Noushmehr H, Salama SR, Zheng S, Chakravarty D, Sanborn JZ, Berman SH, Beroukhim R, Bernard B, et al. The somatic genomic landscape of glioblastoma. Cell. 2013; 155:462–477.
5. Cancer Genome Atlas Research Network. Comprehensive molecular profiling of lung adenocarcinoma. Nature. 2014; 511:543–550.
